# Survey and Validation of tRNA Modifications and Their Corresponding Genes in *Bacillus subtilis* sp Subtilis Strain 168

**DOI:** 10.3390/biom10070977

**Published:** 2020-06-30

**Authors:** Valérie de Crécy-Lagard, Robert L. Ross, Marshall Jaroch, Virginie Marchand, Christina Eisenhart, Damien Brégeon, Yuri Motorin, Patrick A. Limbach

**Affiliations:** 1 Department of Microbiology and Cell Science, University of Florida, Gainesville, FL 32611, USA; mjaroch@ufl.edu; 2 Genetics Institute, University of Florida, Gainesville, FL 32611, USA; 3 Department of Cancer Biology, University of Cincinnati, Cincinnati, OH 45221, USA; rossrb@ucmail.uc.edu; 4 UMR7365 IMoPA CNRS-UL and UMS2008 CNRS-UL-INSERM, Université de Lorraine, Biopôle UL, 54000 Nancy, France; virginie.marchand@univ-lorraine.fr (V.M.); motorine5@univ-lorraine.fr (Y.M.); 5 Department of Chemistry, University of Cincinnati, Cincinnati, OH 45221, USA; psihouca@mail.uc.edu (C.E.); limbacpa@ucmail.uc.edu (P.A.L.); 6 IBPS, Biology of Aging and Adaptation, Sorbonne University, 7 Quai Saint Bernard, CEDEX 05, F-75252 Paris, France; damien.bregeon@sorbonne-universite.fr

**Keywords:** tRNA modifications, model bacteria, Gram-positive, methylation, pseudouridine synthase, YhcT, YjbO

## Abstract

Extensive knowledge of both the nature and position of tRNA modifications in all cellular tRNAs has been limited to two bacteria, *Escherichia coli* and *Mycoplasma capricolum*. *Bacillus subtilis* sp subtilis strain 168 is the model Gram-positive bacteria and the list of the genes involved in tRNA modifications in this organism is far from complete. Mass spectrometry analysis of bulk tRNA extracted from *B. subtilis*, combined with next generation sequencing technologies and comparative genomic analyses, led to the identification of 41 tRNA modification genes with associated confidence scores. Many differences were found in this model Gram-positive bacteria when compared to *E. coli*. In general, *B. subtilis* tRNAs are less modified than those in *E. coli*, even if some modifications, such as m^1^A22 or ms^2^t^6^A, are only found in the model Gram-positive bacteria. Many examples of non-orthologous displacements and of variations in the most complex pathways are described. Paralog issues make uncertain direct annotation transfer from *E. coli* to *B. subtilis* based on homology only without further experimental validation. This difficulty was shown with the identification of the *B. subtilis* enzyme that introduces ψ at positions 31/32 of the tRNAs. This work presents the most up to date list of tRNA modification genes in *B. subtilis*, identifies the gaps in knowledge, and lays the foundation for further work to decipher the physiological role of tRNA modifications in this important model organism and other bacteria.

## 1. Introduction

As adaptors between the mRNA and the elongation peptide, tRNAs are the central decoding elements of the translation process. All tRNA molecules harbor post-transcriptional modifications, and studies from the last 50 years have firmly established the diverse and critical roles that these play in the efficiency and accuracy of translation [1,2]. tRNA modifications can influence tRNA stability and quality control [3,4], they can be determinants or anti-determinants for other components of the translation apparatus such as aminoacyl-tRNA synthetases, and they can restrict or increase the decoding capacity of a given tRNA [5,6]. Since these roles are also modification specific, the effect of inactivating a given tRNA modification enzyme can range from cell death to only subtle phenotypes. For example, t^6^A37 is essential in *E. coli*, most certainly because it is a determinant for IleRS [7]. Lysidine (or k^2^C) is essential in many bacteria because it allows the modified minor tRNA^Ile^_CAU_ to decode AUA codons [8,9,10]. On the other hand, deleting most modification genes only gives rise to subtle fitness defects or to phenotypes which are observed in very specific conditions [1,11].

While the role of tRNA modifications as regulatory devices was proposed in the early 1990s by B.C. Persson [12], it is only recently that this model has gained traction [13,14]. Due to the degeneracy of the genetic code, the same amino acid can be encoded by different codons read by tRNAs with different modification profiles. Hence, if specific genes are enriched in codons decoded by a tRNA that harbors a given modification, changes in the level of this specific modification will modify the proteome. The identification of these Modification Tunable Transcripts (or MoTT) in a wide range of organisms (reviewed in [15]) has led to models for regulation in response to stress or metabolic imbalance. Other regulatory roles for tRNA modifications include their capacity to act as determinants for RNases [3] that can generate regulatory half-tRNA fragments, even if this has mostly been observed in eukaryotes [16,17].

**Figure 1:**
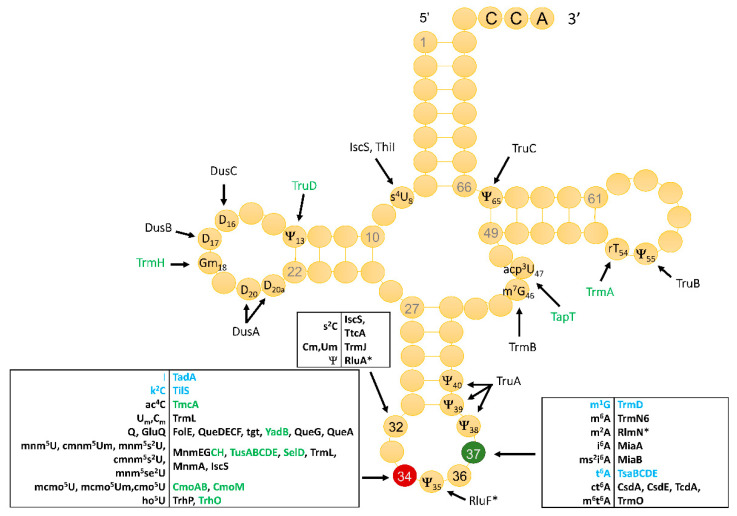
*Escherichia coli* tRNA modifications and corresponding enzymes. Proteins encoded by essential genes are in blue. Proteins with a * also modify rRNAs. The annotations were captured from the Björk and Hargevall review [11], from Uniprot, and the recent literature. In green are proteins with no ortholog in *B. subtilis*. Uniprot IDs for all proteins listed are given Table S1.

To discover these regulatory roles, it is essential to have the foundational knowledge about which tRNA modifications are present in a given organism and which enzymes catalyze their synthesis. Unfortunately, to date, this knowledge is limited to only two bacteria. One is the Gram-positive *Mycoplasma capricolum.* Indeed, because of its small genome size and the pioneer work that generated all tRNA sequences, it was the first bacterium with a full set of predicted tRNA modification genes [18]. The other is the model Gram-negative *Escherichia coli* K12. As shown in Figure 1, decades of biochemical and genetic analyses have identified all the genes involved in the discovered tRNA modifications in this organism. These 59 tRNA modification enzymes and factors represent 1.5% of the *E. coli* encoded proteome.

Recently, a combination of mass spectrometry (MS) and tRNA-Seq methods to detect tRNA modifications was performed on *Vibrio cholerae* [19], and even though this bacteria is closely related to *E. coli*, novel modifications were identified. Full analysis of the modified nucleosides present in tRNAs has been performed for three Gram-positive organisms, *Lactococcus lactis* [20], *Streptomyces griseus* [21], and *Mycobacterium bovis* BCG [22], but the links with the corresponding genes were not made in these studies. *Bacillus subtilis* sp subtilis strain 168 is the model Gram-positive that was sequenced in 1997 [23], and an active community is keeping the genome annotation up to date [24,25]. Even the current knowledge of tRNA modification genes has not been well captured in SubtiWiki, as not all tRNAs have been sequenced. There are some clear gaps in knowledge, which we attempted to fill in the current study by analyzing the modifications found in *B. subtilis* tRNAs, using a combination of MS and tRNA-Seq methods, linking them to the corresponding validated or predicted gene, and experimentally testing a paralog case for the synthesis of the pseudouridines at positions 31 and 32.

## 2. Materials and Methods

### 2.1. Bioinformatic Analyses

The *B. subtilis* genome that was used was NC_000964. The presence and numbers of isoforms were extracted from the GtRNA database (http://gtrnadb.ucsc.edu/GtRNAdb2/genomes/bacteria/Baci_subt_subtilis_168/) [26]. The RNA sequence data were extracted from Modomics (http://modomics.genesilico.pl/sequences/list/?type_field=tRNA&subtype=all&species=4&display=Display&nomenclature=abbrev) [27] from tRNAdb (http://trnadb.bioinf.uni-leipzig.de/) [28] and TpsiC databases (http://tpsic.igcz.poznan.pl/info/start/) [29]. The information on all genes involved in tRNA modification in *E. coli* and *B. subtilis* (Tables S1 and S2) were gathered from the literature and further checked using Subtiwiki [24], Uniprot Id mapping tools [30], and BlastP [31] searches at NCBI [32], as well as Pathosystems Resource Integration Center (PATRIC) feature group tools [33]. The SEED database [34] was used to analyze physical clustering. Sequences were visualized using the integrated genome viewer (IGV) (http://software.broadinstitute.org/software/igv/) [35]. Clustergrammer (http://amp.pharm.mssm.edu/clustergrammer/) was used to visualize fitness and expression data as heatmaps [36]. The Colombos v3.0 database (http://colombos.net/) [37] was used to mine the available *B. subtilis* expression data. Using the list of predicted *B. subtilis* tRNA modification genes from Table S2 as input, an expression matrix comprised of 90 experiments (or contrasts) where these genes showed significant expression was generated from the data available in the database, and further analysis was conducted on this dataset. Each contrast is a single RNA-Seq or microarray experiment where RNA transcript levels for a test condition are compared to a reference culture. For each contrast, Colombos assigns a score that corresponds to how many genes show similar changes in expression. If the changes in expression, relative to their respective reference, increase or decrease uniformly for all the input genes, then a higher score is awarded to the contrast. In the case where a contrast’s tested condition causes the input genes to show dissimilar and noncoherent expression, a lower score is awarded to the contrast. From the list of 90 contrasts, 47 contrasts were selected, with scores less than 0.5, in order to analyze the conditions that caused the input genes to show dissimilar expression. To illustrate drastic changes in expression, 12 genes were selected that showed unique changes in expression values from 4.5 (red) to −5.0 (blue) across the 47 contrasts.

### 2.2. Strains and Media and Bulk tRNA Preparation

*Bacillus subtilis* subsp. subtilis str. 168 (RefSeq: NC_000964.3) was used as the wild-type strain. The mutants used were BKE11620 (*trpC2* Δ*yjbO*::kan) and BKE09210 (*trpC2* Δ*yhcT*::kan) [38]. Cells were grown in Luria–Bertani medium (LB), supplemented with kanamycin (5µg.mL^−1^) at 30 °C, and harvested at an OD (A_600nm_) of 0.6. Bulk RNA was extracted by hot acidic phenol [35] or using chaotropic agents [39].

### 2.3. Mass Spectrometry Analyses

#### 2.3.1. LC-MS of Nucleosides

Digestion of the purified tRNA fraction was performed as previously described [39]. Separation was accomplished by reversed phase chromatography using an Acquity UPLC HSS T3, 1.8 µm, 1 mm × 100 mm column, (Waters, Milford, MA, USA) on a Vanquish Flex Quaternary UHPLC system (Thermo Fisher Scientific, San Jose, CA, USA). Mobile phase A consisted of 5.3 mM ammonium acetate (Thermo Fisher Scientific) in LC-MS grade water (Alfa Aesar, Haverhill, MA, USA), pH 5.3. Mobile phase B consisted of a 60:40 mixture of 5.3 mM ammonium acetate and acetonitrile (Honeywell Burdick & Jackson, Morris Plains, NJ, USA) with a gradient of 0% B (from 0 to 1.8 min), 2% B at 3 to 3.5 min, 3% B at 4.1 min, 5% B at 7 min, 25% B at 9 min, 35% B at 15 min, 99% B at 15.5 min (hold for 4.5 min), and 99% B at 20 min, then returning to 0% B at 25.5 min, at a flow rate of 100 µL min^−1^. The column temperature was set to 40 °C. Accurate mass values and physio-chemical properties were determined using Marvin 17.3.13.0 (https://chemaxon.com).

High resolution accurate mass (HRAM) analysis was performed on an Orbitrap Fusion Lumos Tribrid mass spectrometer (Thermo Fisher Scientific) interfaced with an H-ESI electrospray source in positive polarity mode. Full scan data were acquired at a resolution of 120,000, mass range 220–900 *m*/*z*, AGC 7.5e4, and IT 100 ms. Data-dependent top speed MS/MS spectra (1 s cycle, CID 40%) were acquired in the ion trap with rapid scan rate, AGC 1.0e5, and an injection time of 300 ms, with scan range mode set to auto *m*/*z* normal. The other instrumental conditions were as follows: quadrupole isolation of 1 *m*/*z*; RF 35%; sheath gas, auxiliary gas, and sweep gas of 30, 10, and 0 arbitrary units, respectively; ion transfer tube temperature of 289 °C; vaporizer temperature of 92 °C; and spray voltage of 3500 V. Data were analyzed using Xcalibur 4.0, Compound Discoverer 3.0, and mzVault 2.1 (Thermo Fisher Scientific). Fragment Ion Search (FISh) settings were set at a high mass accuracy of 2.5 ppm, a low mass accuracy of 0.5 Da, and a S/N of 5000.

#### 2.3.2. LC-MS Analysis of Oligonucleotides

Separation was accomplished by HILIC chromatography, using a Shodex HILICPak VN-50 2D, 5 µm, 2 mm × 150 mm column, (Showa Denko, New York, NY, USA) on a Vanquish UHPLC system (Thermo Fisher Scientific, San Jose, CA, USA). Mobile phase A consisted of 50 mM ammonium bicarbonate (Sigma Aldrich, St. Louis, MO, USA) in 18 Ohm millipure water, pH 9.0. Mobile phase B consisted of acetonitrile (Honeywell Burdick & Jackson, Morris Plains, NJ, USA) 0.1% NH_4_OH, with a gradient of 30% B for 1.5 min, increasing to 35% B at 17 min, 70% B at 30 min, and holding for 2 min, then returning to 30% B at 32.1 min and re-equilibrating to 45 min at a flow rate of 200 µL min^−1^. The column temperature was set at 20 °C.

RNase T1 digestion products were analyzed using the Orbitrap Fusion Lumos Tribrid mass spectrometer (Thermo Fisher Scientific) interfaced with an H-ESI electrospray source in negative polarity mode. Data-dependent top speed MS/MS spectra (1 s cycle, CID 40%) were acquired in the ion trap with rapid scan rate, AGC 1.0e5, and an injection time of 300 ms. Data-dependent top speed MS/MS spectra (1 s cycle, CID 40%) were acquired in the ion trap with rapid scan rate, AGC 1.0e5, and an injection time of 300 ms. The other instrumental conditions were as follows: quadrupole isolation of 1.6 *m*/*z*; RF 30%; sheath gas, auxiliary gas, and sweep gas of 40, 8, and 1 arbitrary units, respectively; ion transfer tube temperature of 275 °C; vaporizer temperature of 320 °C; and spray voltage of 2500 V. Data were analyzed using Xcalibur 4.0 and RAMM 1.0 [40].

### 2.4. Identification of Modification by Next Generation Sequencing Methods

Deep sequencing-based identification of the *B. subtilis* tRNA modifications was performed using RiboMethSeq protocol, allowing us to map 2′-O-methylations by their protection to cleavage [41,42]. Reverse Transcriptase (RT) misincorporation signatures at RT-arresting nucleotides [43,44] were extracted from RiboMethSeq data and also from the RT-primer extension, using TTO-based ScriptSeq v2 (Illumina, San Diego, CA, USA) library preparation kit. RiboMethSeq analysis of tRNAs was performed using alkaline fragmentation of total *B. subtilis* RNA, followed by library preparation and sequencing [42]. RT misincorporation signatures were extracted from Samtools mpileup format and manually validated by inspection of aligned reads in *.bam file in IGV. Pseudouridine mapping (Psiseq) was performed in the tRNA-enriched fraction of *B. sublitis* RNAs, using a chemical-based protocol derived from CMCT (1-cyclohexyl-(2-morpholinoethyl)carbodiimide metho-p-toluene sulfonate)-chemical mapping protocol, and specifically adapted to tRNAs [45,46,47].

## 3. Results and Discussion

### 3.1. Identity and Position of tRNA Modifications in the Model Gram-Positive B. subtilis 168

**Figure 2:**
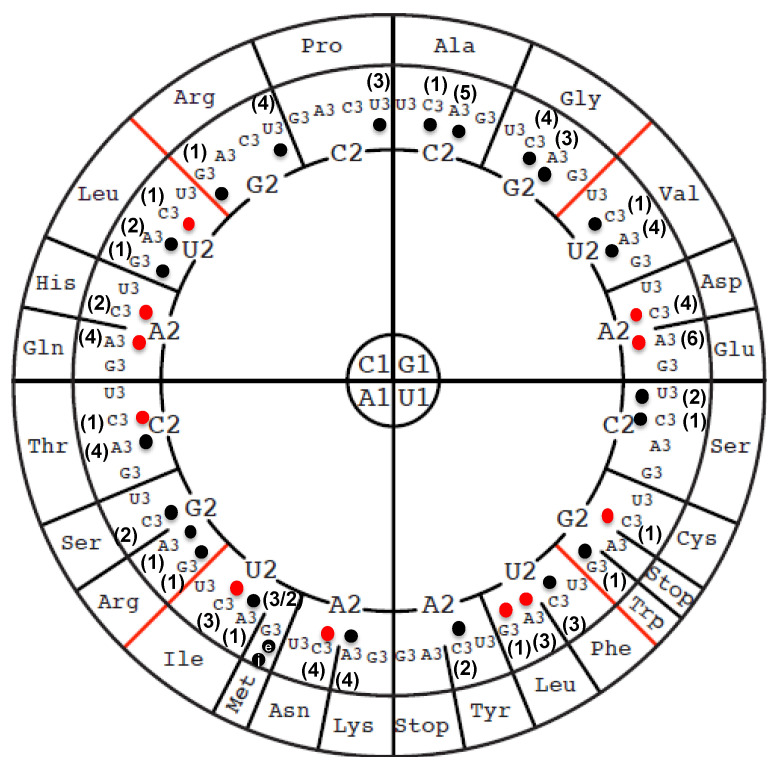
Compilation of sequenced *B. subtilis* tRNA genes and tRNA molecules. Circular representation of the genetic code displaying modified nucleosides based on Grosjean and Westhof [48]. The codon sequence of each amino acid is read in the inside-out direction (1-2-3). The numbers of isoforms are shown by the number in parentheses and the data were extracted from the GtRNA-db 2.0 [26]. A red dot specifies that the tRNA has not been sequenced, while a black dot specifies that the tRNA sequence is available based on the information available in Modomics [27]. The “e” and “i” specify the elongator or initiator tRNA^Met^_CAU_, respectively.

A total of 86 tRNA genes specifying 35 different iso-acceptors can be identified in the *Bacillus subtilis* 168 genome (Figure 2). The RNA sequences of 24 of these have been reported (black dots in Figure 2), but 11 tRNA isoacceptors remain to be sequenced (red dots in Figure 2). To fill this knowledge gap, different methods were combined. First, a comprehensive MS analysis of the bulk tRNA extracted from *B. subtilis* cells was performed. These data were complemented by analysis of the RT-arresting nucleotides and 2′-O-methylation by RiboMethSeq, as well as pseudouridines by PsiSeq.

Two types of LC-MS/MS analyses were performed. The first consisted of a total nucleoside identification assay, where an aliquot of the bulk tRNA is taken to its nucleoside monomers by enzymatic digestion to identify which modifications are present in the sample within the detection limits. In the second assay, an aliquot of the bulk tRNA is digested with base specific nucleases to create oligonucleotides that can be separated chromatographically by length and whose fragmentation ladder allows the placement of the modifications detected in the nucleoside assay into their proper sequence context. For modification mapping by LC-MS/MS, an initial survey scan was conducted using lower resolution mass analyzers (i.e., ion trap and q-TOF), and these data provided a rough map to guide higher resolution analyses conducted on the orbitrap. The mass spectrometry data reported here resulted from final verification using the orbitrap, with a few exceptions that are noted.

Next Generation Sequencing(NGS)-based analysis of the tRNA modifications (and, eventually, rRNA modifications) was performed using three complementary techniques. First, analysis of the RT-arrests and misincorporation profiles was performed using the TTO-based Illumina library preparation kit. However, random priming during the RT-primer extension step makes this kit more suitable for long RNAs (like rRNAs) and not for short tRNAs, thus these data were only used as a complement. The second and major approach used in the study was standard RiboMethSeq, initially developed for 2′-O-methylation detection. We also noticed that, in addition to 2′-O-methylations, moderate RiboMethSeq signals were frequently detected also for pseudouridines, dihydrouridines (D), and m^7^G residues, present in tRNAs. These data were used only as supplementary confirmation of known sites. In addition, the same RiboMethSeq datasets were used for the detection of RT-misincorporations. Direct ligation of 3′- and 5′-adapters to RNA or RNA fragments makes this approach unsuitable for the detection of hard RT-stops, but strong RT misincorporation profiles (e.g., Inosine, m^1^A, m^3^U, m^,6,2^A, m^2,2^G, s^4^U, etc.) were still detected, albeit with a somewhat lower efficiency due to the robustness of SuperScript III, which was used in the RT step. The third deep sequencing-based approach used a modified pseudouridine-specific chemical modification protocol and allowed the detection of candidate sites in the tRNAs. A summary of the RiboMethSeq and PsiSeq analyses of the *B. subtilis* tRNAs is given in Table S3.

Thirty-five modified ribonucleosides were identified in the bulk purified tRNA by LC-MS/MS (Table 1). Of these 35, 21 had previously been mapped in *B. subtilis*, as listed in Modomics or in additional studies [49,50]. Several modifications (epoxyQ (oQ), t^6^A, mnm^5^U, and nm^5^U) are intermediates in complex modification pathways [51,52,53]. Another two (2′-O-methylations Cm and U*m) were only detected by RiboMethSeq. Several modified ribonucleosides must result from rRNA contamination copurifying with the tRNA during anion exchange fractionation [39]. These include m^4^Cm, m^6,2^A, m^2^G, m^2,2^G, and Am. m^4^Cm is not found in any tRNA but is present at position 1402 of *E. coli* 16S rRNA [54]. This modification is conserved in other Gram-positive bacteria [55], with *B. subtilis* encoding the orthologs of the *E. coli* RmsH/RsmI enzymes involved in m^4^Cm1402 synthesis (BSU_00360 and BSU_00360, respectively). The contaminating rRNAs were abundant enough that they could be analyzed by NGS, and a candidate site for this rRNA modification (C*m) in *B. subtilis* rRNA was detected by RiboMethSeq, in a highly conserved sequence at position 1410 (equivalent to *E. coli* position 1402) (Figure S1). m^6,2^A is another 16S rRNA modification, found at two adjacent adenosines (A1518 and A1519, *E. coli* numbering), that is very conserved in rRNA but not found in tRNAs, and it is introduced by the KsgA enzyme [56,57] that is present in *B. subtilis* (BSU_00420). Both m^6,2^A residues are visible by their RT-signatures at the corresponding positions A1527 and A1528 in *B. subtilis* 16S rRNA, as shown Figure S1. Two other modifications detected in the MS analysis of bulk nucleosides were also attributed to rRNA contamination. The methylated guanosines m^2^G and m^2,2^G are found in archaeal and eukaryotic tRNAs, where they have structural roles [58,59,60], but they have never been identified in bacterial tRNAs. Both are found in bacterial rRNAs [55,61]. An ortholog of *E. coli* RsmD involved in m^2^G966 formation in 16S rRNA is present in *B. subtilis* (YlbH/BSU_15010). In *Clostridium acetobutylicum* and *Thermus thermophilus*, this position is actually modified to m^2,2^G [55], so this could also be the case in *B. subtilis*. In addition, an ortholog of *E. coli* RlmKL that is involved in m^2^G2445 in the formation 23S rRNA was identified in *B. subtilis* (YpsC/BSU_22170). We were able to tentatively map the m^2,2^G modification to CpG dimers (Cp[m^2,2^G]p) at the following positions: G285 in 16S rRNA and G2884 in 23S rRNA (Figure S1). Finally, the Am modification could also come from an rRNA modification, since a number of candidate Am sites were found (positions 224 and 1455 in 16S rRNA as well as 1075, 1524, 2220, and 2827 in 23S rRNA) (Figure S1).

Several observed modifications must be artifacts of the sample preparation. m^1^I has only been observed in archaeal tRNA [62] but could arise from the chemical or enzymatic deamination of m^1^A during the enzymatic digestion to nucleosides [63]. Similarly, oxidation inside the cell or during analysis is most certainly the source of 8-oxoG formation [64].

The base position cannot be determined for simple methylations by oligonucleotide mass mapping; for example, m^1^A, m^2^A, and m^6^A are all detected as only a methylated adenosine. We were able to map an adenosine methylation at position 37 on tRNA^Met^CAU, as well as ac^4^C at position 34 (Figure S2A). The sequence of the *E. coli* equivalent tRNA suggests that the probable modification is m^6^A. A methylated adenosine was also mapped to position 22 of the tRNA^Leu^ UAA (Figure S2B). This adenosine was identified as m^1^A, based on prior literature [65], and this was confirmed by RT-signature analysis (Figure S1 and Table S3). We also mapped the m^2^A found at position 37 of tRNA^Glu^ UUC, having mnm^5^s^2^U at U34 (Figure S2C). The presence of m^1^A previously reported at position 58 of tRNA^Leu^ CAG [66] was never reproduced (Louis Droogmans, personal communication) and was not detected in our analyses. The unknown C32 modification in tRNA^Lys^TTT reported by Vold et al. [67] was not detected by either the LC-MS or the RiboMethSeq approach.

In addition, we mapped mnm^5^s^2^U (Figure S2D), as well as mnm^5^U (Figure S2E) and cmnm^5^s^2^U (Figure S2F), to different tRNAs (Table 1). The modification 5-methoxyluridine (mo^5^U) is found in a number of *E. coli* tRNAs at position 34. Here, we were able to map mo^5^U position 34 of tRNA^Leu^ UAG (Figure S2G). Moreover, 4-thiouridine was mapped to position 8 of tRNA^Gln^UUG by MS (Figure S2H) and of tRNA^Leu^UAA by RT (Figure S1). The hypermodification ct^6^A, along with the t^6^A counterpart, was found in tRNA^Thr^ UGU (Figure S2I,J) and tRNA^Ile^ GAU (Figure S2K,L) at position 37. Queuosine was mapped to its normal position 34 in tRNA^His^GUG and tRNA^Asp^ GUC. (Figure S2M,N) with spectral complexity hindering the mapping of tRNA^Tyr^ and tRNA^Asn^. Finally, the modification inosine was mapped to position 34 of the tRNA^Arg^ACG (Figures S1 and S2O). Due to either low abundance or spectral complexity we were unable to map all the modifications detected through nucleoside mass spectrometric analysis to an oligonucleotide sequence. For example, the modification cmnm^5^s^2^Um and ms^2^t^6^A were mapped to the anticodon of tRNA^Leu^UAA, however the complexity of the MS/MS spectra makes this assignation tentative. The presence of 2′-O-methylation at the modified U*34 in tRNA^Leu^UAA was also confirmed by RiboMethSeq (Figure S1). Mapped modification position for those which were detected are listed in Table 1.

### 3.2. Compilation of Predicted and Validated B. subtilis tRNA Modification Genes

**Figure 3:**
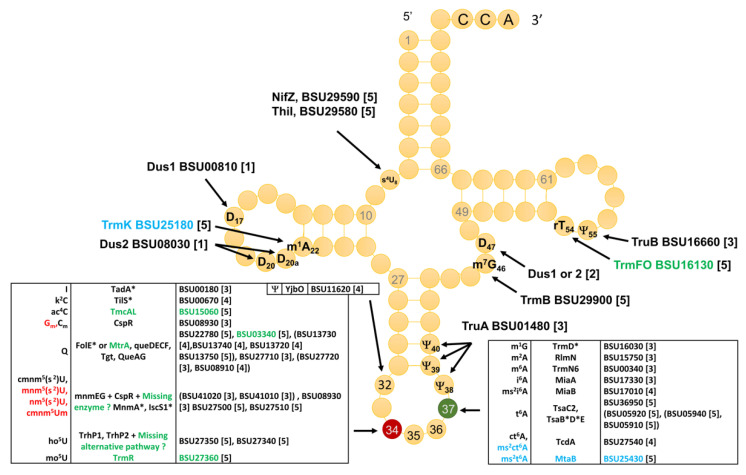
*B. subtilis* tRNA modifications with predicted and validated corresponding enzymes. The four modifications in red were found in the current analysis but were not present in the *B. subtilis* tRNA sequences listed in Modomics. Proteins with stars (*) are encoded by essential genes in *B. subtilis*. Proteins in green are non-orthologous to the enzyme catalyzing the same reaction in *E. coli*. Proteins in blue insert modifications that are not found in *E. coli*. The confidence score for the prediction is as follows: (5), validated in vitro with the *B. subtilis* enzymes and in vivo with the *B. subtilis* deletion mutant; (4), orthologous to a validated enzyme in other species, genetic data in *B. subtilis*; (3), orthologous to a validated enzyme in other species, with mapping data in *B. subtilis*; (2), orthologous to a validated enzyme in other species, without mapping data in *B. subtilis*; (1), similarity with experimentally enzyme validated in other species but paralogs.

Combining literature searches with sequence similarity searches using the set of known enzymes of *E. coli* as input, and crossing these data with the list of tRNA modification genes in SubtiWiki [24], led to a list of tRNA modification genes in *B. subtilis* (Figure 3 and Table S3). The level of confidence for the various predictions was given by a score that ranged from (5) to (1) (see Figure 3 legend for the full description of the scoring system). This analysis led to the identification of 41 predicted tRNA modification genes in *B. subtilis* that will be discussed in more detail below.

### 3.3. The Majority of tRNA Modifications Are Introduced by Orthologs in E. coli and B. subtilis with Some Differences Observed in the Complex Pathways

Many enzymes of the tRNA modification machinery are conserved between *E. coli* and *B. subtilis*. Indeed, out of the 41 proteins predicted to be involved in inserting tRNA modifications into the Gram-positive model, the majority (32/41) are clear orthologs of *E. coli* proteins. These include proteins involved in the synthesis of I34 (TadA), k^2^C34 (TilS), m^1^G37 (TrmD), i^6^A (MiaA) ms^2^i^6^A37 (MiaB/YmcA), ψ38/39 (TruA), m^7^G46 (TrmB), ψ55 (TruB), and m^6^A37 (TrmN/YabB), for which there is also experimental evidence of the presence of the modifications at these positions in the tRNA (Table 1). For RlmN/YloN, no tRNA sequence containing this modification is found in Modomics, but our analysis revealed a possible m^2^A at position 37 in tRNA^Glu^UUC (see discussion above and Figure S2C).

For the enzymes involved in ribose methylation at position 34, the *B. subtilis* CspR is an ortholog of the *E. coli* TrmL, but U*m/Cm are found in *E. coli* tRNAs, while U*m/Gm/Cm were identified in the *B. subtilis* tRNAs by RiboMethSeq. We confirmed the 2′-O-methylation of G_m_34 in tRNA^Phe^GAA and identified C_m_34 in tRNA^Leu^CAA and U*m34 in tRNA^Gln^UUG and tRNA^Leu^UAA (Figure S1). No signals corresponding to the 2′-O-methylation inserted in *E. coli* by the TrmH and TrmJ enzymes (Gm at position 18 and Um/Cm at position 32, respectively) (Figure 1) were identified in the *B. subtilis* tRNAs (data not shown).

In the cases of most multi-step pathways, such as those involved in s^4^U34, Q34, t^6^A37, and xm^5^s^2^U34 syntheses, many small variations can be found between the two model organisms; even the enzymes involved in the core machineries of theses pathways are conserved. The Q synthesis pathways are identical in *E. coli* and *B. subtilis*, with every step catalyzed by an orthologous enzyme. The only differences are that an additional enzyme (FolE2) can catalyze the first step in *B. subtilis* [69] and there is no ortholog of the GluQRS/YadB enzyme in *B. subtilis* [70]. The t^6^A37 synthesis core machineries are also very similar in the two model organisms, with differences found both at the beginning and end of the pathways. The enzymes that catalyze the first step are slightly different: TsaC in *E. coli* [71,72] and TsaC2, which carries an extra SUA5 domain, in *B. subtilis* [71,73]. In addition, different t^6^A hypermodifications are found in the two organisms. Whereas m^6^t^6^A is present in *E. coli* [74], ms^2^t^6^A is found *B. subtilis*, synthesized by the MiaB paralog MtaB [75,76]. An ortholog of the *E. coli* ct^6^A formation enzyme TcdA was validated in *B. subtilis* and both ct^6^A and ms^2^ct^6^A can be detected in this organism [68], even if we detected only ct^6^A in the current study (Table 1). It is not clear if additional sulfur transfer proteins are required in *B. subtilis*, like they are in *E. coli* (CsdAE) [53]. The xm^5^U derivatives identified in the *B. subtilis* tRNAs (Table 1) are similar to those present in *E. coli,* as confirmed by the Armengod laboratory [49]. The exception is cm^5^s^2^U, which might be an intermediate as the full synthesis of these complex modifications varies greatly depending on the media conditions [49]. Orthologs of two of the three *E. coli* enzymes involved in synthesizing this complex modification (MnmE, MnmG) are found in *B. subtilis.* However, no MnmC ortholog is present, an observation already made by the Armengod group, who also showed that another enzyme catalyzing the same activity must be present and that it does not require S-adenosyl-L-methionine [49]. There are also differences in the thiolation enzymes which synthesize the xs^2^U34 derivatives. If the enzyme that introduces the sulfur in tRNA, MnmA, is similar in both organisms, *E. coli* uses a whole sulfur relay system (TusABCDE) [77], while *B. subtilis* uses only one relay enzyme of the IscS family, YrvO/IscS1 [78,79]. Finally, in *E. coli*, the ribose methylase required to synthesize cmnm^5^Um is the multifunctional TrmL; it is therefore parsimonious to propose that, similarly, CspR catalyzes this reaction in *B. subtilis*, even though this has not been confirmed experimentally.

### 3.4. Modifications and Enzymes Specific to Bacillus subtilis

In addition to ms^2^t^6^A, another modification that is present only in *B. subtilis* and not *E. coli* is m^1^A22. This modification is inserted by the TrmK gene, which was described over 10 years ago [80] and is a marker of Gram-positive bacteria, as it is even found in those bacteria with minimal genomes, such as *M. capricolum* [18]. G_m_34 in tRNA^Phe^GAA (Table S3) is also found in *B. subtilis* but not *E. coli* and is reminiscent of eukaryotic tRNA modification profiles [81,82,83].

Moreover, beyond the MnmC discussed above, where a non-homologous enzyme, still to be discovered, must catalyze the same reaction in *B. subtilis*, several cases of non-orthologous replacement are found between the two model organisms. The first is the well-studied replacement of the SAM dependent *E. coli* methylase TrmA (COG2265) by the *B. subtilis* folate dependent enzyme TrmFO (COG1206) in order to synthesize m^5^U54 [84]. Another case has recently been discovered. In the synthesis of ac^4^C34, the *E. coli* TmcA acetyltransferase of the COG1444 family, which uses acetyl-coA donor for the acetyl group [85], has been replaced by the TmcAL (COG1323) protein in *B. subtilis*, which uses acetate and ATP to create an acetyl-AMP intermediate as the acetyl donor in different mechanisms [50].

The two other non-orthologous displacement examples are slightly more complex. Both *E. coli* and *B. subtilis* harbor the s^4^U8 modification, but the modification enzymes are slightly different [78,86]. The *B. subtilis* ThiI is shorter than the *E. coli* homolog, lacks the rhodanese domain, and is not involved in thiamin synthesis. In addition, Gram-positive organisms harbor dedicated cysteine desulfurase for specific thiofactor generation, whereas *E. coli* uses the generalist IscS for all of them. NifZ is the one dedicated cysteine desulfurase that is involved in s^4^U synthesis in *B. subtilis* [86]. Finally, different enzymes modify the ho^5^U precursor in order to give different xmo^5^U34 derivatives. In *E. coli*, the two CmoA and CmoB enzymes synthesize cmo^5^U34 in a complex reaction that uses a S-adenosyl-S-carboxymethyl-l-homocysteine (Cx-SAM) intermediate [87]; the cmo^5^U34 intermediate can be further modified to mcmo^5^U, and then mcmo^5^Um, by CmoM and TrmL, respectively [88]. In *B. subtilis*, a classical methyltransferase (TrmR) directly methylates ho^5^U34 to mo^5^U34 [89], and no orthologs of CmoM or TrmL are found. The enzymes involved in ho^5^U synthesis have been identified recently [90]. In *E. coli*, TrhP (formerly known as YegQ), a peptidase U32 family protein, is involved in prephenate-dependent ho^5^U formation, while TrhO (formerly known as YceA), a rhodanese family protein, catalyzes oxygen-dependent ho^5^U formation and bypasses cmo^5^U biogenesis in a subset of tRNAs under aerobic conditions. In *B. subtilis*, the TrhP homologs TrhP1/YrrN and TrhP2/YrrO are also involved in one pathway, but another pathway has also been predicted and the corresponding genes have not yet been identified [91].

### 3.5. Paralog Issues Make Direct Annotation Transfers Problematic for a Few Modifications

There are several cases in which the homologs of validated *E. coli* tRNA modification enzymes are found in *B. subtilis* but, because of the paralog issue, it is not possible to give a high confidence functional annotation without additional experiments. This is the case for the identification of the *B. subtilis* dihydrourine insertion enzymes. In *E. coli*, DusC modifies position 16, DusB modifies position 17, and DusA modifies positions 20 and 20a [92]. The three proteins are members of the same COG0042 family. Dihydrouridine is found at four different positions in *B. subtilis* tRNAs: D17, D20, D20a, and D47 (Figure 3). However, only two COG0042 members are encoded in the *B. subtilis* genome: Dus1 (BSU00810) and Dus2 (BSU08030). At this stage, without further biochemical and/or genetic experiments, it is not possible to predict which position each enzyme modifies.

**Figure 4:**
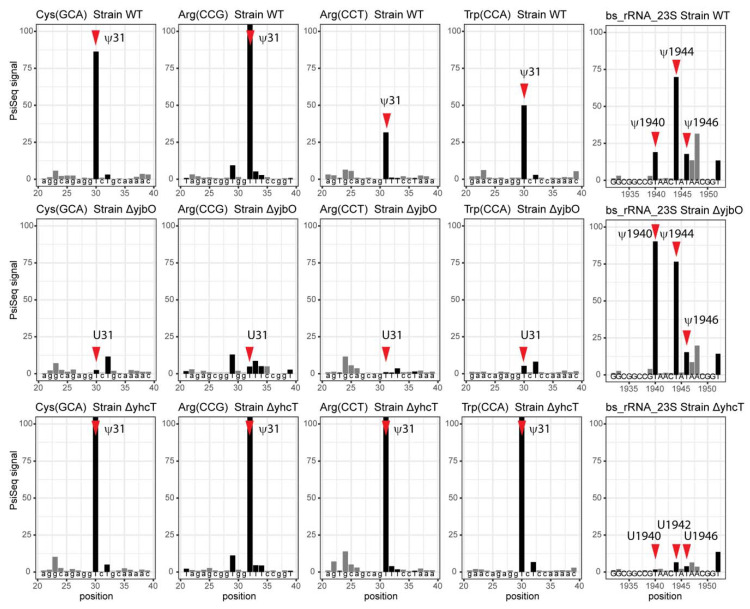
Analysis of pseudouridine residues in tRNAs and rRNAs by PsiSeq. Normalized PsiSeq signals are shown for selected regions in *B. subtilis* tRNAs and 23S rRNA. The top row corresponds to the wild-type strain, the middle one for the Δ*yjbO* mutant, and the bottom one for the Δ*yhcT* mutant. Positions of known and detected pseudouridine residues are shown by red arrows. Conventional numbering of tRNA positions may not correspond to real position of nucleotide in the sequence due to missing residues and inclusion of 17a, 20a, 20b, and variable loop nucleotides. Identity of tRNA and its anticodon are indicated on the top of each panel. YjbO is involved in pseudouridylation of position 31 (and also 32) in several tRNAs. YhcT is involved in pseudouridylation of 23S rRNA positions 1940, 1944, and 1946 and is the functional equivalent of *E. coli* RluD.

Another case is the prediction of the *B. subtilis* pseudouridine synthase involved in the synthesis of ψ31/32 catalyzed in *E. coli* by RluA, an enzyme that modifies both rRNA and tRNA substrates. Searching the *B. subtilis* genome, using *E. coli* RluA as an input and using BlastP with default settings, identified the two RluA homologs YhcT and YjbO, both of which are COG0564 members. Here, again, it was impossible to predict if these proteins had the same substrate specificity as the *E. coli* RluA, or if they had each evolved to modify just tRNA or rRNA or just one position (31 or 32), or if they had another function in the cell. We therefore analyzed by Pseudo-Seq the bulk tRNA preparations extracted from *B. subtilis* strains lacking each gene, respectively. As shown in Figure 4 and Figure S3, the absence of YjbO led to the absence of ψ at position 31 in tRNA^Trp^CCA, tRNA^Cys^GCA, tRNA^Arg^CCG, and tRNA^Arg^CCU, while these were not affected by the absence of YhcT. Similar results were observed for the presence of ψ at position 32 in tRNA^Arg^CCU and tRNA^Pro^UGG (Figure S3), suggesting that YhbO was responsible for modifying both the 31 and 32 positions in the tRNAs.

Since YhcT did not seem to be involved in tRNA modification, we tested whether it could be modifying rRNA. Previous studies from the Ofengand laboratory [93,94] mapped ψ modifications in the 23S rRNAs of *E. coli* and *B. subtilis* and identified the corresponding *E. coli* genes. As shown in Table 2, 10 residues are modified to ψ in *E. coli* 23 S rRNA, but only four of those are also modified in *B. subtilis.* It was previously shown that the functional homolog of RluB in *B. subtilis* was YpuL/BSU23160, which was actually closer in sequence to the *E. coli* enzyme RsuA that modifies the small subunit [95]. PsiSeq analysis suggests that YhcT is the functional homolog of *E. coli* RluD and modifies positions 1940, 1944, and 1946 of the 23S rRNA, even if it is closer in sequence to RluA (Figure 4).

This leaves one pseudouridine synthase to be identified for the modification of position 2521(2520) in the 23S rRNA in *B. subtilis*. The equivalent position in *E. coli* (U2492) is not modified. A plausible candidate is the third COG0564 *B. subtilis* member, YlmL (BSU15460), a prediction that requires experimental validation. As a side note of this analysis, no ortholog of *E. coli* RluF was identified in *B. subtilis,* and in *E. coli*, this enzyme also modifies position 35 of tRNA^Tyr^QΨA [96]. We therefore predict that the corresponding *B. subtilis* tRNA is not modified with ψ at this same position, and this is confirmed by the PsiSeq analysis (Table S3).

### 3.6. System Analysis of tRNA Modification Genes in B. subtilis

As *B. subtilis* is such a prominent model organism, many whole genome datasets are available. These include mutant collections, phenotypic screens, TnSeq datasets, and gene expression data [24]. Many of the tRNA modification genes are poorly annotated and/or were only recently discovered, so we revisited several published genome-wide studies that used 42 *B. subtilis* tRNA modification genes as input.

Seven out of the 42 *B. subtilis* modification genes are essential (Figure 3) [38]. *FolE/mtrA* is essential because of its role in the synthesis of the tetrahydrofolate cofactor, but not its role in Q synthesis, as it can be replaced by *folE2/yciA* only under zinc limitation [69]. *TilS* is essential in most bacteria [10], with the rare exception of organisms such as *Mycoplasma mobile* that have changed the minor tRNA^Ile^ anticodon from CAU to UAU, making the lysidine modification dispensable [97]. The essentiality of *trmD* in many bacteria has already made it an antibacterial target [98]. Interestingly, two t^6^A37 synthesis genes are essential (*tsaB* and *tsaD*), whereas two others (*tsaC* and *tsaE*) are not, while all four orthologs are essential in *E. coli* [7]. This suggests that the essentiality of *tsaBD* in *B. subtilis* could be due to another role in the cell besides t^6^A formation in the tRNA. Finally, the *iscS*1 and *mnmA* involved in s^2^U34 formation are essential in *B. subtilis* but not in *E. coli* [99], for reasons that are not totally clear.

**Figure 5:**
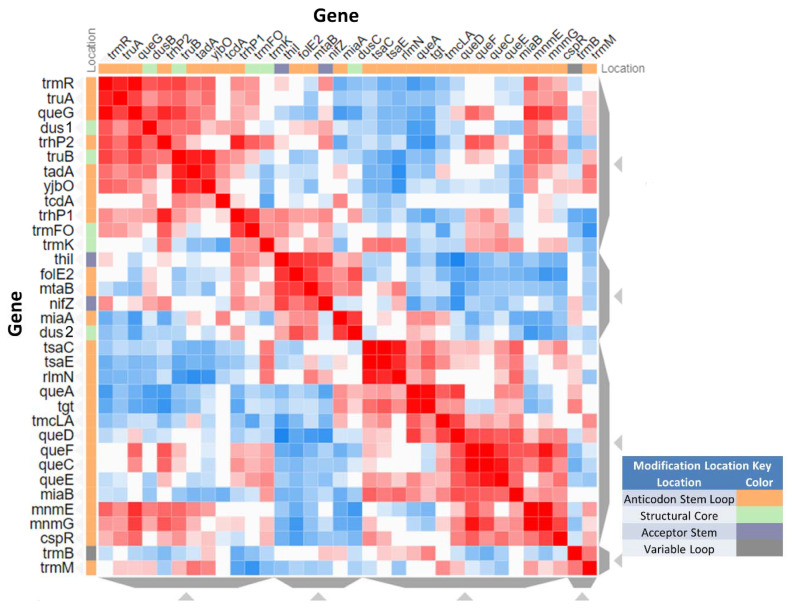
Fitness Data Similarity Matrix for predicted tRNA modification genes. The fitness scores for the ErmR mutant set are displayed as a similarity matrix, with the deleted gene shown on each of the axes of the matrix. The position of the tRNA modification that each gene mediates is denoted by the colored box next to each gene’s name. Fitness scores with similar values are shown in red while dissimilar scores are shown in blue. The full dataset is given in Table S4.

A phenotype screen testing several conditions (growth, C or N sources, and temperature sensitivity) of two ordered, barcoded, erythromycin-resistance- and kanamycin-resistance-marked single-gene deletion libraries is available [38]. Fitness scores are given for every gene in every condition tested. We extracted from these data the scores for the 35 predicted tRNA modification genes that are not essential (Table S4), and we visualized these data using clustergrammer, after transforming them into Z-scores. While transforming the fitness data into Z-scores may have exaggerated the differences between the ErmR and KanR datasets, it was noted in their original publication [40] that discrepancies between datasets could be caused by the downstream effects of the resistance cassettes that were used to prepare each library of mutants. Since we observed differences in the fitness data between the ErmR and KanR datasets for our list of genes, only the ErmR dataset was used to prepare Figure 5. Any phenotype observations from these datasets should be experimentally validated.

As shown in Figure 5, genes in same modification pathways, such as *tsaC*/*tsaE* (t^6^A37), *tgt*/*queA* (Q), *queDCEF* (Q precursor), and cmnm^5^U34 (*mnmE/G*), have similar or the same fitness profiles, suggesting a biological relevance that will need to be further studied.

We also looked at published *B. subtilis* Tn-Seq studies and found that three tRNA modification genes have been identified as being involved in sporulation (*mnmE*, *mnmG*, and *miaA*) [100], and *mnmG* was also important for germination [101].

**Figure 6:**
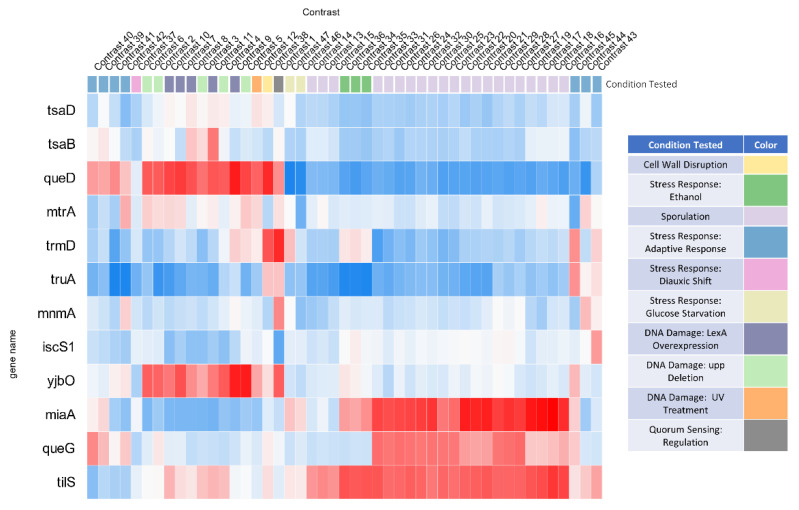
Most dramatic observed expression changes for tRNA modification genes. A subset of genes with dramatic shifts in expression levels across 47 contrasts were selected to create a separate heatmap. Genes with increased expression levels are shown in red and genes with decreased expression levels are shown in blue. The gene names are shown on the left axis and the contrasts are listed on the top axis. See Table S5 for the complete data and the lists of conditions.

Using the list of locus tags as input, we analyzed the expression of 42 *B. subtilis* RNA modification genes in all transcriptomic data available in the Colombos database (41 tRNA modification and one involved in rRNA modification) [37] (Table S5). Some conditions lead to reductions or increases in most tRNA modification genes and are logically conditions where all translation genes would be expected to be affected, such as those that affect growth rate or transcription inhibition. To further explore the conditions where the response was specific to just a few genes, we focused on the conditions that led to differences in expression for just a subset of genes, as described in the Methods section. As shown in Figure 6, we found that twelve genes involved in the synthesis of Anticodon Stem Loop modifications (ψ31/32, Q34, ψ38/39, i^6^A37, t^6^A37, k^2^C34, s^2^U34) were up or down regulated in specific stress conditions, opening possible avenues for further exploration.

## 4. Conclusions

This study strengthens the efficiency of using an integrated strategy to identify tRNA modifications and the corresponding genes in a given organism, as recently illustrated with *V. cholerae* [19]. Mass spectrometry, tRNA-Seq, and comparative genomic methods do not give accurate or complete results on their own, but when they are used in combination, one can generate a high confidence tRNA modification map. Such integrated approaches involving both LC-MS analysis and the deep sequencing-based mapping of RNA modifications will be particularly useful for the analysis of still unexplored bacterial, archaeal, and eukaryotic organisms.

Even though we still have not been able to sequence all tRNA molecules of *B. subtilis*, and hence some modifications might remain to be to discovered and/or mapped, we do believe that the set of tRNA modification genes in *B. subtilis* is nearly complete, with only two predicted genes missing (the functional homolog of *mnmC* and an alternate ho^5^U synthesis gene) (Figure 3 and Table S3). The model Gram-positive *B. subtilis* harbors slightly fewer modifications (2/3) than the model Gram-negative *E. coli* but contains twice the amount found in the minimal Gram-positive *M. capricolum* (Table 3).

As shown in Figure S4, the levels of specific tRNA modifications can vary greatly between rare modifications such as k**^2^**C34 or abundant modifications such as i**^6^**A37. Further studies will be required to follow how modification levels change with growth conditions or stresses, but the transcriptomics and phenotype data do suggest that the levels of specific modifications are affected by growth conditions and that cellular processes that integrate tRNA modifications could be part of the regulatory loop in *B. subtilis*.

## Figures and Tables

**Table 1:** Modified ribonucleosides identified in *B. subtilis* tRNAs.

Detected in Bulk Nucleoside Analysis and Mapped by RiboMethSeq	Previously Mapped	Mapped by MS	Mapped by NGS	Pathway Intermediate
**tRNA modifications**				
1-methyladenosine, m^1^A	M^%^	Glu22 UUC^1^ Tyr22 GUA^1^	Cys22 GCA Gln22 UUG Glu22 UUC Leu22 CAA Leu22 UAA Leu22 GAG Tyr22 GUA Ser22 GGA Ser22 GCT Ser22 UGA	
1-methylguanosine, m^1^G	M	Pro37 UGG Leu37 CAG		
2-methyladenosine, m^2^A		Glu37 UUC		
2-methylthio-N6-isopentenyladenosine, ms^2^i^6^A	M	Leu37 UAA		
2-methylthio-N6-threonylcarbamoyladenosine, ms^2^t^6^A	M^2^	Ser37 UGA		
2’-O-methylcytidine, Cm			Leu34 CAA	
2’-O-methylguanosine, Gm	M		Phe34 GAA	
Modified U* with 2’-O-methyluridine, U*m			Lys34 UUU	
Modified U* with 2’-O-methyluridine, U*m			Gln34 UUG	
4-thiouridine, s^4^U	M	Gln8 UUG	Leu8 UAA Gln8 UUG Tyr8 GUA	
5-aminomethyluridine, nm^5^U				P [49]
5-carboxymethylaminomethyl-2′-O-methyluridine, cmnm^5^Um	P^&^ [49]	Leu34 UAA	Leu34 UAA	
5-carboxymethylaminomethyl-2-thiouridine, cmnm^5^s^2^U	M/[49]	Gln34 UUG		
5-methoxyuridine, mo^5^U	M	Leu34 UAG		
5-methylaminomethyl-2-thiouridine, mnm^5^s^2^U	[49]	Glu34 UUC Gln34 UUG		
5-methylaminomethyluridine, mnm^5^U		Gln34 UUG		[49]
5-methyluridine, m^5^U	M			
7-methylguanosine, m^7^G	M			
cyclic N6-threonylcarbamoyladenosine, ct^6^A	M^2^	Leu37 UAA Ile37 GAU Thr37 UGU		
Dihydrouridine, D	M	Asp21-22 GUC Ile21-22 GAU Ile21-22 CAU	X	
Epoxyqueuosine, oQ				X
Inosine, I	M	Arg34 ACG	Arg34 ACG	
Lysidine, k^2^C	M			
N4-acetylcytidine, ac^4^C	[50]	Met34 CAU		
N6-isopentenyladenosine, i^6^A	M			
N6-methyladenosine, m^6^A	M	Met37 CAU		
N6-threonylcarbamoyladenosine, t^6^A		Ile37 GAU Thr37 UGU		M^3^
Pseudouridine, ψ	M		many, ψ 31/32/55	
Queuosine, Q	M	His34 GUG Asp34 GUC		
**rRNA modifications**				
2-methylguanosine, m^2^G				
N4,2’-O-dimethylcytidine, m^4^C_m_			16S rRNA 1410	
N2,N2-dimethylguanosine, m^2,2^G			putative	
N6,N6-dimethyladenosine, m^6,6^A		Yes	16S rRNA1527/28	
2’-O-methyladenosine, Am			putative	
**Chemical or biochemical artifacts**				
1-methylinosine, m^1^I				
8-oxoguanosine, oxoG				

^%^M, listed in Modomics; ^&^P, predicted. ^1^Note 1: m^1^A found in low resolution mapping data but not independently verified in high resolution data. ^2^Note 2: ms^2^ct^6^A37 previously reported [68] but not detected here. ^3^Note 3: t^6^A is an intermediate but is also found when the tRNA is prepared in standard conditions and hence this is the molecule reported in Modomics.

**Table 2:** Comparison of ψ sites and corresponding modification enzymes in 23S rRNA of *E. coli* and *B. subtilis*.

E.c. Mod	B.s. Mod (n° in Ref [93,94])	E.c Protein	B.s Protein	Evidence
Ψ746	U793 (791)	RluA	NA	
Ψ955	U1001 (999)	RluC	NA	
Ψ1911	Ψ1940 (1938)	RluD	YhcT/BSU09210	PsiSeq
Ψ1915 *	Ψ1944 * (1942)	RluD	YhcT/BSU09210	PsiSeq
Ψ1917	Ψ1946 (1944)	RluD	YhcT/BSU09210	PsiSeq
Ψ2457	U2486(2484)	RluE	NA	
U2492	Ψ2521 (2520)	NA	YlmL/BSU15460	Only sequence similarity
Ψ2504	U2533 (2532)	RluC	NA	
Ψ2580	U2609 (2608)	RluC	NA	
Ψ2604	U2633 (2632)	RluF	NA	
Ψ2605	Ψ2634 (2633)	RluB	YpuL/BSU23160	Experimental [95]

* Certainly further modified to m^3^ψ, as attested by RT signature for this position (Figure S1).

**Table 3:** Comparison of numbers of tRNA modifications and associated genes in three model organisms.

Organism	Modifications at Specific Positions in tRNAs	Modification Genes
*Bacillus subtilis* subsp. subtilis str. 168	35	41 + 2 missing
*Mycoplasma capricolum*	17	22
*Escherichia coli* K12 MG1655	45	59

## Supplementary Materials

The following are available online at https://www.mdpi.com/2218-273X/10/7/977/s1, Table S1: Validated *E. coli* tRNA modification genes, Table S2: Predicted and validated *B. subtilis* modification genes, Table S3: Summary of B. subtilis tRNA modification mapping by RiboMethSeq and PsiSeq, Table S4: Fitness data for tRNA modification genes in *B. subtilis*, Table S5: Expression data tRNA modification genes in *B. subtilis*, Figure S1: Mapping of tRNA and rRNA modification by RiboMethSeq and derived RT-signatures, Figure S2: Tandem mass spectrum of different tRNAs at different positions, Figure S3: Bar plots for normalized PsiScore values for six *B. subtilis* tRNAs containing ψ31 and ψ32 residues, Figure S4: Relative ion abundances of tRNA modifications found in *B. subtilis* 168.
