# Supplementary material for: Survey and Validation of tRNA Modifications and Their Corresponding Genes in Bacillus subtilis sp Subtilis Strain 168
Source: Biomolecules. 2020 Jun 30;10(7):977. doi: 10.3390/biom10070977 (PMC7408541; doi:10.3390/biom10070977)
Supplement: Supplementary file 1 [file biomolecules-10-00977-s001.zip › SupDat_proofs/FigureBs-tRNAmod_FIG_S1_S4.pdf]

# Figure S1

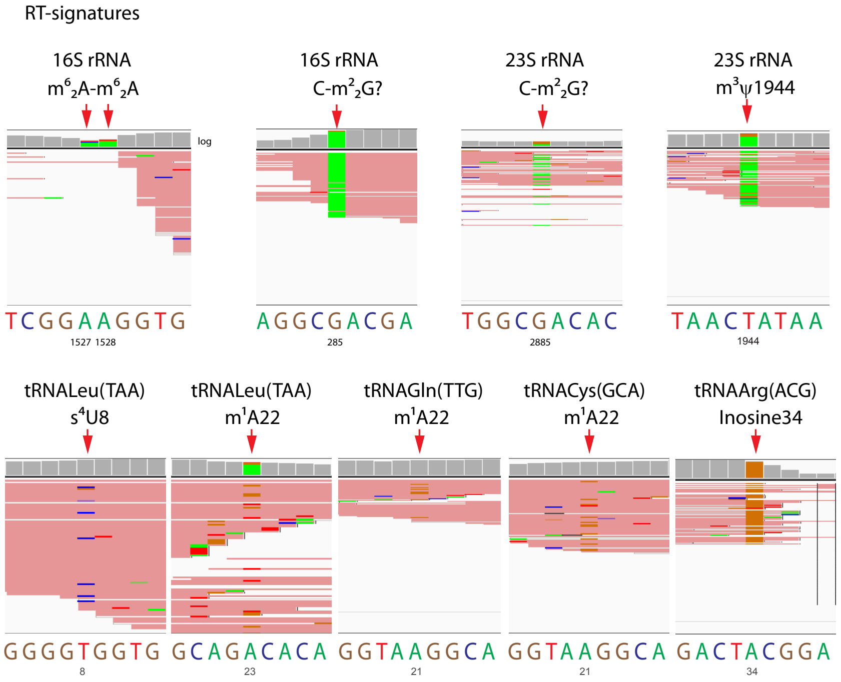


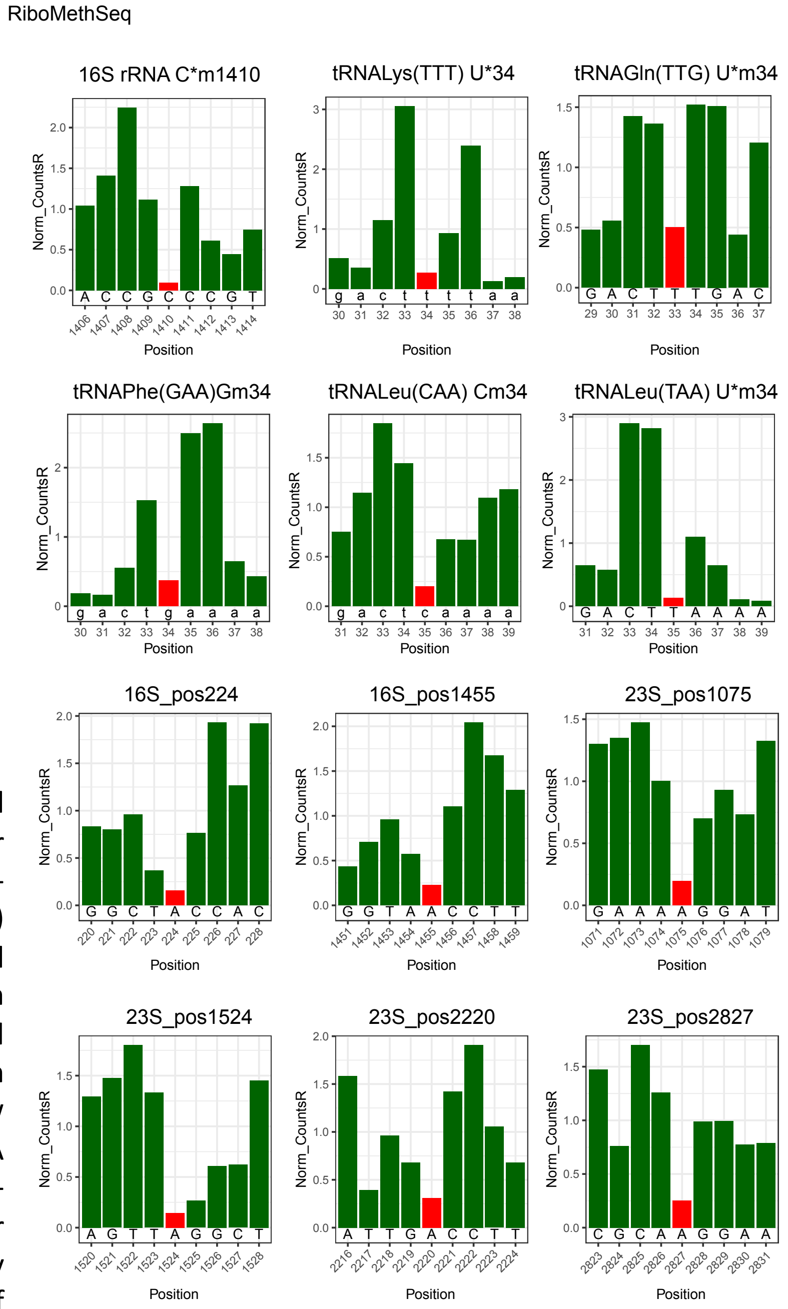


**Fig. S1**. Mapping of tRNA and rRNA modification by RiboMethSeq and derived RT-signatures. RT signatures are visualized by IGV and shown for selected positions in B. subtilis rRNAs (16S rRNA m$^{62}$A1527/1528, putative C-m$^{22}$G dinucleotides in 16S and 23S rRNAs and m$^{3}$y1944 residue in 23S rRNA) and tRNAs (LeuUAA-s$^{4}$U8, m$^{1}$A22, GlnUUG-m1A22, CysGCA-m$^{1}$A22 and ArgACG-I34). Sequence and numbering is indicated at the bottom of each panel. Conventional numbering of tRNA positions may not correspond to real position of nucleotide in the sequence due to missing residues and inclusion of 17a, 20a, 20b and variable loop nucleotides. RiboMethSeq data show locally normalized cleavage-protection profile observed for selected rRNA and tRNA positions. Norm_CountsR values were calculated as cumulated 5'- and 3'-end read count for a given position, normalized to median value for the region. Candidate site is indicated in red. Backshift of -1 nt was already introduced in the protection profile, the gap corresponds to the position of modified residue. Sequence and numbering are shown at the bottom. Identity of RNA and position are shown on the top of each panel.

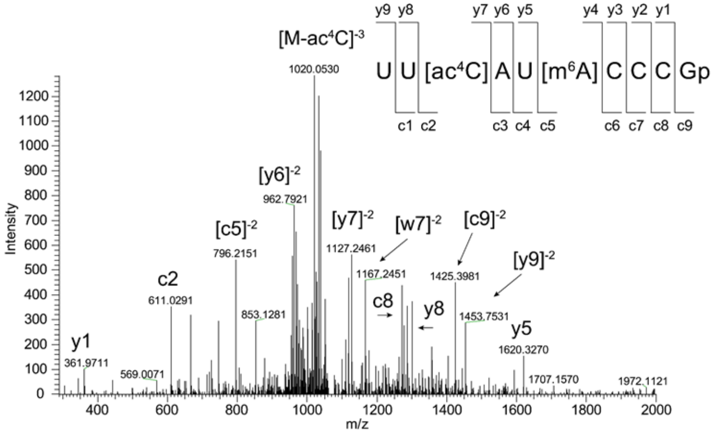


Fig S2A. Tandem mass spectrum of the anticodon tRNA[Met] CAU showing presence of the modifications $ac^4C$ at position 34 and $m^6A$ at position 37.

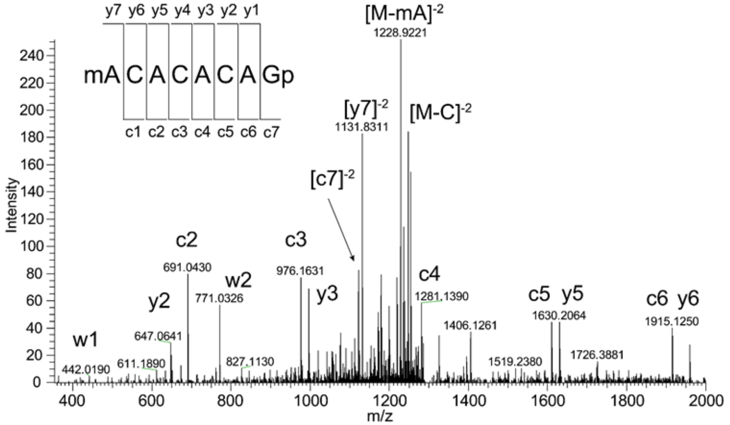


Fig S2B. Tandem mass spectrum of the position 22 of the tRNA[Leu] UAA. Prior literature and RNAseq analysis suggest the methylation is $m^1A$.

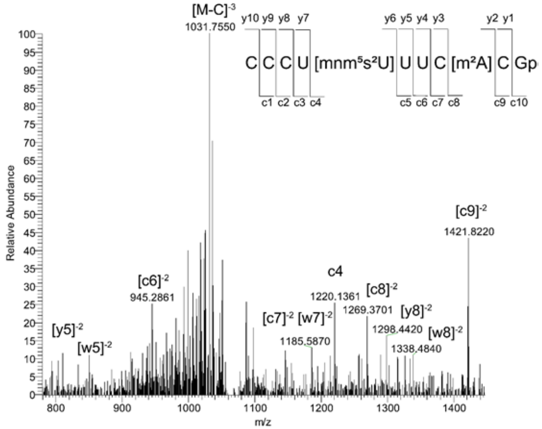


Fig S2C. Tandem mass spectrum of the anticodon tRNA[Glu] UUC showing presence of the modification $mnm^5s^2U$ and position 34 and $m^2A$ at position 37.

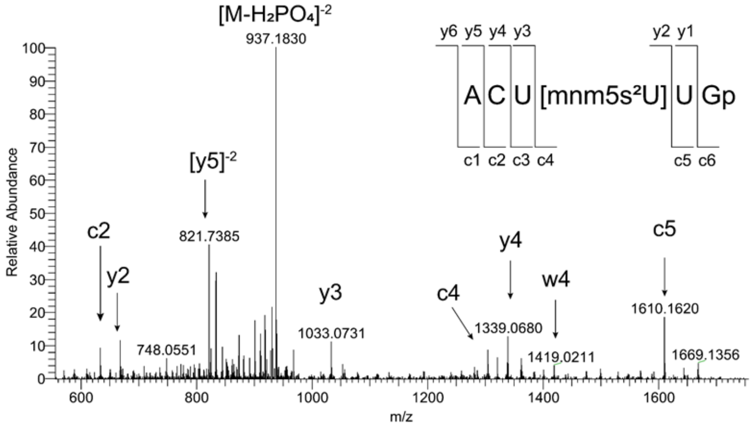


Fig S2D. Tandem mass spectrum of the anticodon tRNA[Gln] UUG showing presence of the modification $mnm^5s^2U$ at position 34.

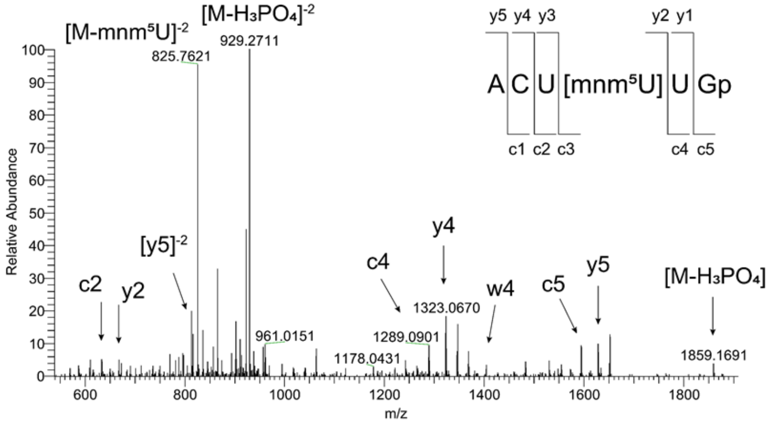


Fig S2E. Tandem mass spectrum of the anticodon $tRNA^{Gln}$ UUG showing presence of the modification $mnm^5U$ at position 34.

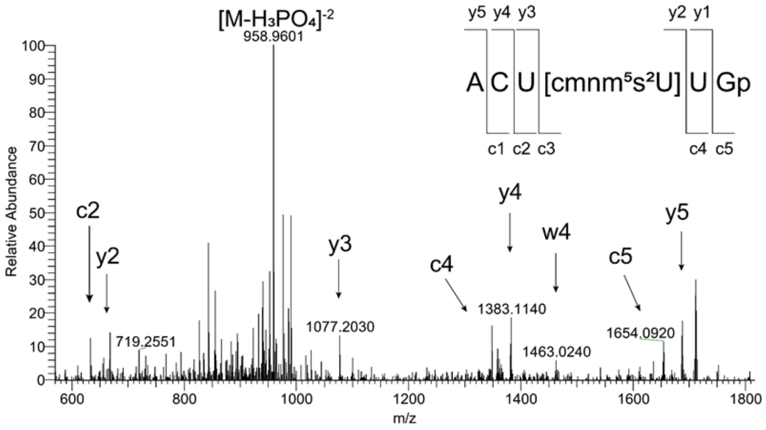


Fig S2F. Tandem mass spectrum of the anticodon $tRNA^{Gln}$ UUG showing presence of the

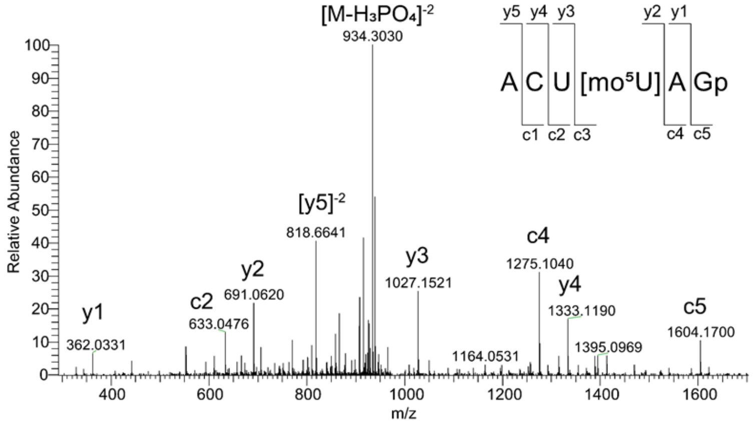


Fig S2G. Tandem mass spectrum of the anticodon $tRNA^{Leu}$ UAG showing presence of the modification $mo^5U$ at position 34.

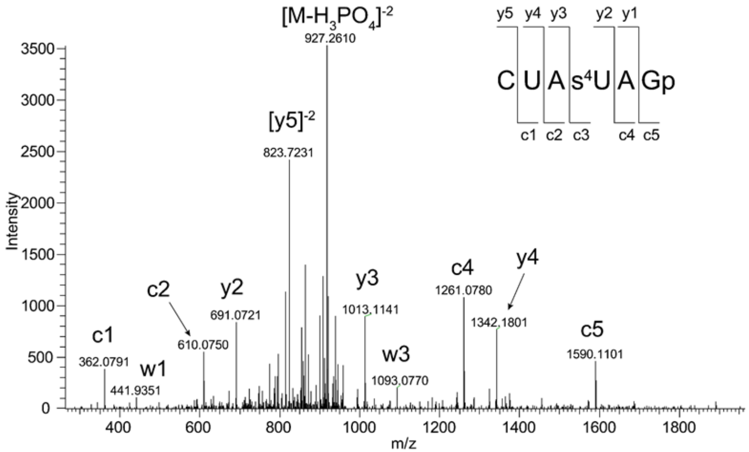


Fig S2H. Tandem mass spectrum of position 8 of $tRNA^{Gln}$ UUG showing presence of the modifications $s^4U$.

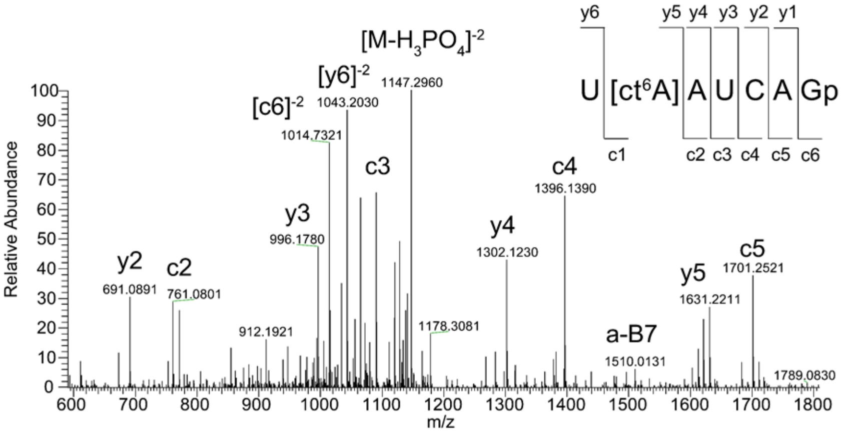


Fig S2I. Tandem mass spectrum of the anticodon in tRNA$^{Thr}$ UGU showing presence of the modifications ct$^6$A at position 37.

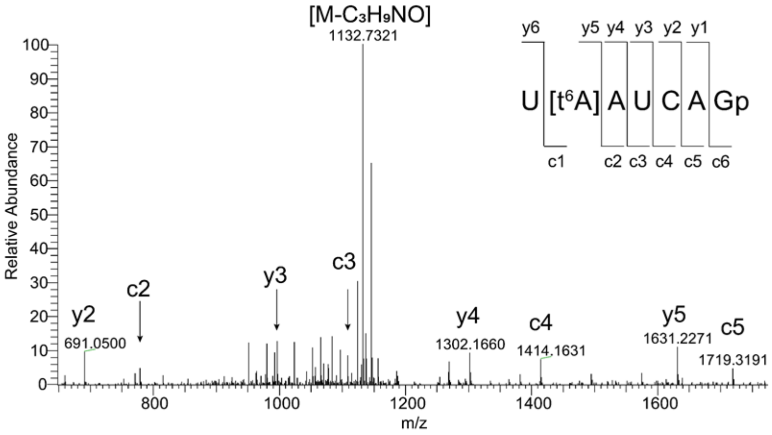


Fig S2J. Tandem mass spectrum of the anticodon in tRNA$^{Thr}$ UGU showing presence of the modifications t$^6$A at position 37.

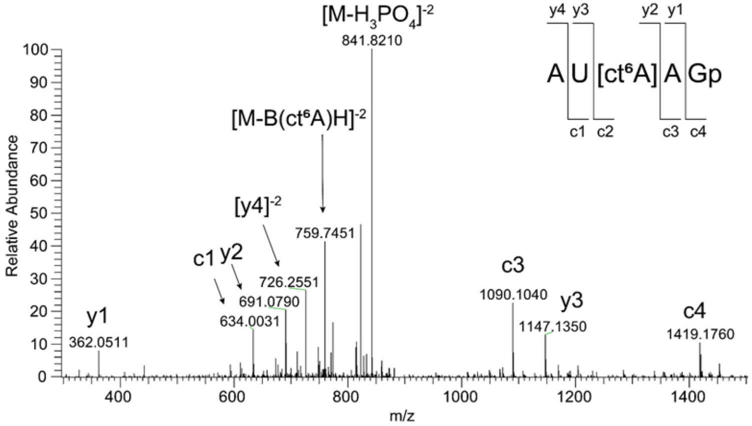


Fig S2K. Tandem mass spectrum of the anticodon in tRNA$^{Ile}$ GAU showing presence of the modifications ct$^6$A at position 37.

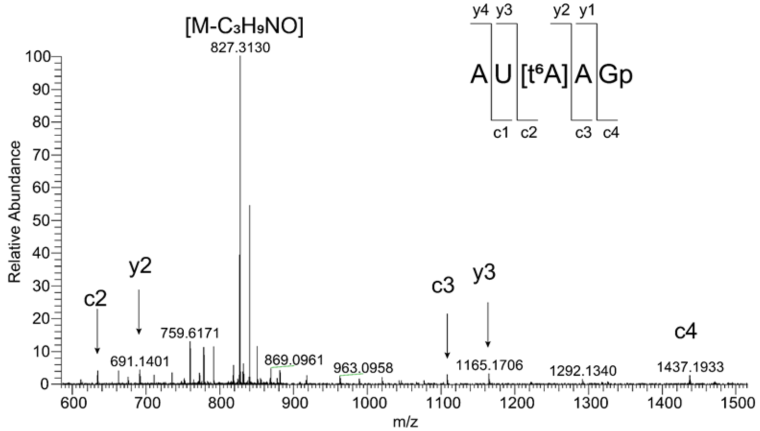


Fig S2L. Tandem mass spectrum of the anticodon in tRNA$^{Ile}$ GAU showing presence of the modifications t$^6$A at position 37.

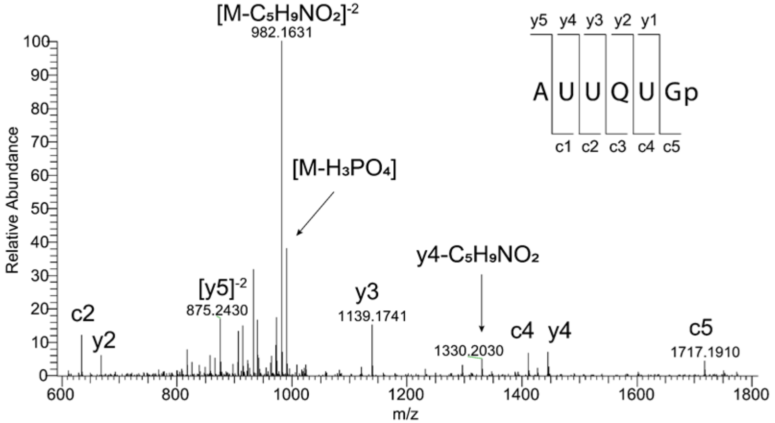


Fig S2M. Tandem mass spectrum of the anticodon in tRNA$^{His}$ GUG showing presence of the hyper-modifications Q at position 34.

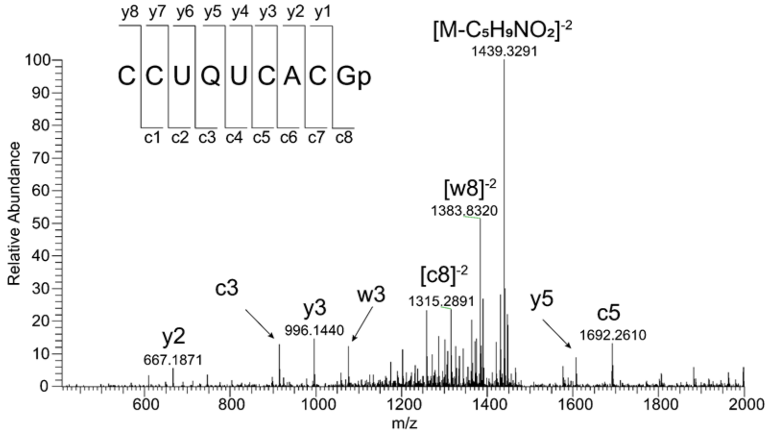


Fig S2N. Tandem mass spectrum of the anticodon in tRNA$^{Asp}$ GUC showing presence of the hyper-modifications Q at position 34.

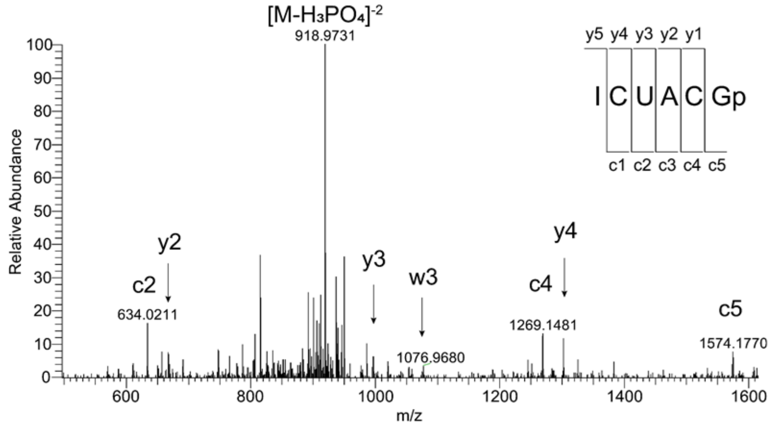


Fig S2O. Tandem mass spectrum of the anticodon in tRNA$^{Arg}$ ACG showing presence of the modifications inosine at position 34.

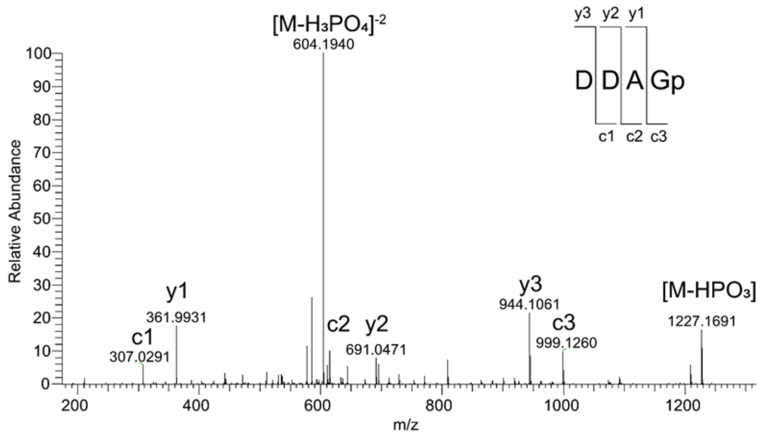


Fig S2P. Tandem mass spectrum of position 21 and 22 in the tRNA$^{Ile}$ GAU and tRNA$^{Ile}$ CAU showing presence of two dihydrouridines.

## Figure S3

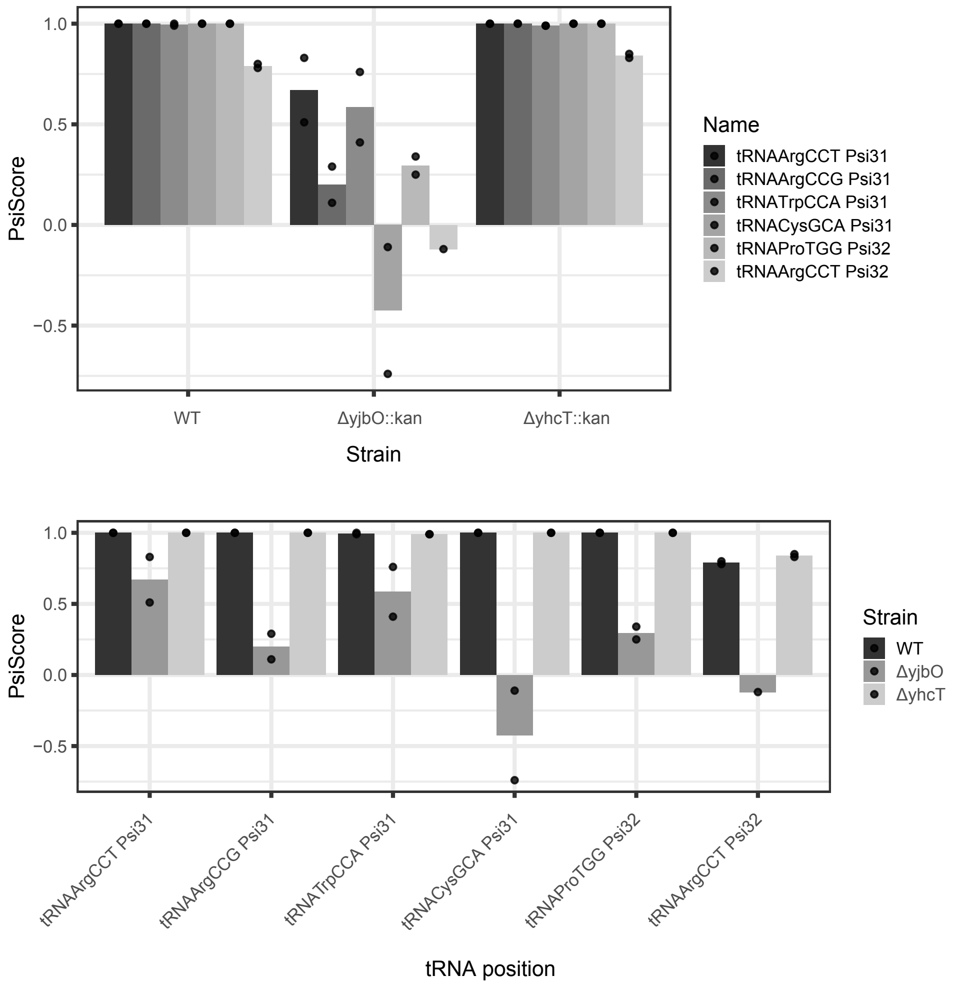


**Fig. S3.** Barplots for normalized PsiScore values for six *B. subtilis* tRNAs containing ψ31 and ψ32 residues. Psi-Scores are shown for WT, ΔyjbO and ΔyhcT strains, grouped either by strain, or by tRNA position. Identity of strain and tRNA analyzed is shown at the side and bottom of the barplot.

**Figure S4**

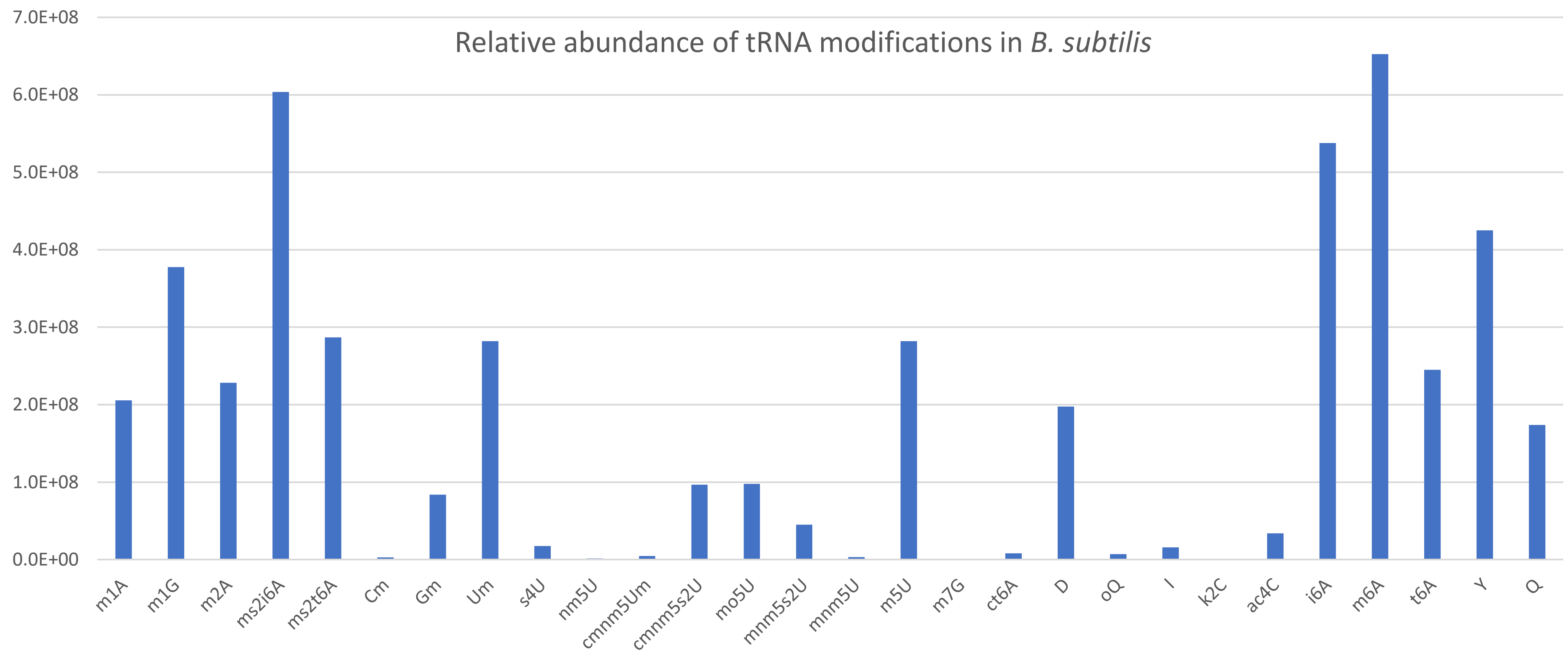


**Figure S4**, Relative ion abundances of tRNA modifications found in *B. subtilis* 168 by HRAM-LC-MSMS. Abundances were determined from integrated peak areas using Xcalibur 4.0 with 15 point gaussian smoothing with Genesis peak detection algorithm. Monoisotopic masses for extracted ion profiles were generated using Marvin 17.3.13.0 (www.chemaxon.com) from the empirical formulas tabulated in Modomics (https://iimcb.genesilico.pl/modomics/)
